# Improving the Insulating Capacity of Polyurethane Foams through Polyurethane Aerogel Inclusion: From Insulation to Superinsulation

**DOI:** 10.3390/nano12132232

**Published:** 2022-06-29

**Authors:** Beatriz Merillas, Fernando Villafañe, Miguel Ángel Rodríguez-Pérez

**Affiliations:** 1 Cellular Materials Laboratory (CellMat), Condensed Matter Physics Department, Faculty of Science, University of Valladolid, Campus Miguel Delibes, Paseo de Belén 7, 47011 Valladolid, Spain; 2 GIR MIOMeT-IU Cinquima-Química Inorgánica, Faculty of Science, University of Valladolid, Campus Miguel Delibes, Paseo de Belén 7, 47011 Valladolid, Spain; fernando.villafane@uva.es; 3 BioEcoUVA Research Institute on Bioeconomy, University of Valladolid, 47011 Valladolid, Spain

**Keywords:** polyurethane foam, polyurethane aerogel, pore size, composites, mechanical properties, thermal superinsulation, Knudsen effect

## Abstract

A novel synthesis of polyurethane foam/polyurethane aerogel (PU_F_–PU_A_) composites is presented. Three different polyurethane reticulated foams which present the same density but different pore sizes (named S for small, M for medium, and L for large) have been used. After the characterization of the reference materials (either, foams, and pure aerogel), the obtained composites have been characterized in order to study the effect of the foam pore size on the final properties, so that density, shrinkage, porous structure, mechanical properties, and thermal conductivity are determined. A clear influence of the pore size on the density and shrinkage was found, and the lowest densities are those obtained from L composites (123 kg/m^3^). Moreover, the aerogel density and shrinkage have been significantly reduced through the employment of the polyurethane (PU) foam skeleton. Due to the enhanced mechanical properties of polyurethane aerogels, the inclusion of polyurethane aerogel into the foam skeleton helps to increase the elastic modulus of the foams from 0.03 and 0.08 MPa to 0.85 MPa, while keeping great flexibility and recovery ratios. Moreover, the synthesized PU_F_–PU_A_ composites show an excellent insulating performance, reducing the initial thermal conductivity values from 34.1, 40.3, and 50.6 mW/(m K) at 10 °C for the foams S, M, and L, to 15.8, 16.6, and 16.1 mW/(m K), respectively. Additionally, the effect of the different heat transfer mechanisms to the total thermal conductivity is herein analyzed by using a theoretical model as well as the influence of the measurement temperature.

## 1. Introduction

Polyurethanes have been widely employed in different sectors as a result of their competitive cost-effectiveness in combination with their versatility and engaging properties. The construction sector has been one of the main fields in which polyurethane has been used as sealant, in adhesives, and as a foam in architectural walls and thermal insulation [1]. Polyurethane foams can be classified according to their cellular structures into closed-cell and open-cell foams. Considering their mechanical strength, polyurethane foams can be divided into rigid or flexible foams, as well as the hybrid form labeled as semirigid. In general, rigid polyurethane foams (PUR) are composed of closed cells in which the gaseous phase is isolated by the pore walls. Their thermal conductivities lie in the range of 20–30 mW/(m K) [2,3,4], and, for this reason, rigid polyurethane foams have been commonly used as thermal insulators in buildings and appliances. However, nowadays these thermal conductivity values are too high and are not in compliance with the building energy requirements [5] and, therefore, new materials with a superior thermal insulating performance are being sought.

Incorporating additives to the polyurethane formulation has been one of the most employed strategies to improve the PUR insulating capacity due to the concomitant reduction of the cell size and reduction of the heat transfer due to radiation. A great variety of fillers have been added to the initial formulations, such as montmorillonite-based organo-nanoclays [6,7], minerals [8], carbon nanotubes [9,10], carbon nanofibers [11], or even aerogels. Different types of aerogel particles have been proven to enhance the PUR foam insulation, as well as the mechanical properties or flame retardancy, such as graphene aerogels [12], alumina aerogels [13], phenolic aerogels [14], or the most commonly used silica aerogels [15,16,17]. Nevertheless, this strategy entails several drawbacks, such as the difficulty to reach a high degree of nanoparticles dispersion, the aggregates formation, or the alterations in the reaction kinetics. The latter effect was studied in 2018 by Santiago-Calvo et al. [18] by analyzing the effect of the addition of different nanoclays and nanosilicas in PU foams by in situ FTIR spectroscopy. The study concluded that isocyanate conversion, as well as the amount and ratio of generated urea and urethane groups, were correlated with the nanoparticles’ surface chemical groups. Therefore, the presence of additives modifies the reaction kinetics during the reactive foaming process, resulting in an additional alteration of the cellular morphology, density, and final properties.

More recently, in 2020, Cimavilla-Román et al. [19] analyzed the effect of silica aerogel particles on the foaming process and on the cellular structure of rigid PU foams by in situ X-ray radioscopy, in situ FTIR spectroscopy, and measurements of the reaction temperature. A delay on the foam expansion and an enhancement on the cell nucleation were observed, as well as changes on the chemical reaction balance during the polymerization and foaming process.

Considering these precedents, the addition of nanofillers to PU foams might promote multiple and simultaneous alterations on the polyurethane kinetics and properties that are not thoroughly under control. In addition, even if a proper dispersion of the particles and a significant cell size reduction and a decrease in the heat transfer by radiation is achieved, the potential reduction of the total thermal conductivity is limited to values not larger than 15–20%.

The idea of improving PU foam properties without altering its formation process is a logical conclusion taking into account the previous contributions. For instance, Maddalena et al. improved the stiffness and flame retardancy of PU foams by deposition of graphite oxide (GO) nanoplates [20]. Several attempts were carried out by Ye et al. [21] and Liu et al. [22] by including silica aerogel into carbon foams, and in fact, excellent thermal properties were reached. However, the strength of the obtained composites, owing to the poor mechanical properties of silica aerogel, could be improved.

Herein, we report the synthesis of novel composites in which polyurethane reticulated foams (a special type of flexible foams in which the polymer forms a continuous network of struts and ribs) are filled with an organic aerogel. The selected organic aerogel was polyurethane aerogel [23], due to its promising properties and to the expectable good interaction with the PU foam. The obtained composites showed lower densities and shrinkages than the monolithic PU aerogel while keeping excellent insulation capacities as well as high mechanical strength, in comparison to the initial foams. The PU foam skeleton prevents the PU aerogel from a strong shrinkage contributing to a reduction in its density from 129.10 to 97.60 kg/m^3^. Moreover, the produced composites presented a great elasticity, being able to recover their initial height after several consecutive compressive tests with a maximum strain of 10%. Finally, the key purpose of the strategy herein described is to reduce the thermal conductivity of the initial PU foams, reaching a reduction of 53, 58, and 68% with final values ca. 16 mW/(m K); therefore, the composites can be considered as superinsulating materials. The development of these improved superinsulating materials, which also present enhanced mechanical properties, opens a wide range of potential applications in the construction, automotive, aeronautical, energy conservation, and energy storage sectors.

## 2. Materials and Methods

### 2.1. Materials

Polyisocyanurate–polyurethane aerogels were synthesized by using pentaerythritol as polyol (purity > 98%) (ρ = 1.396 g/cm^3^ at 20 °C) provided by Alfa Aesar (Thermo Fisher GmbH, Kandel, Germany). The employed isocyanate was a polymeric methylene diphenyl diisocyanate (p-MDI) (4,4’-diphenylmethane diisocyanate) commercialized as IsoPMDI^®^ 92,140 (ρ = 1.23 g/cm^3^ at 25 °C) by BASF Polyurethane (Ludwigshafen, Germany). KOSMOS^®^ 75 MEG supplied by Evonik (Essen, Germany) was used as catalyst. The catalyst solution (70 g/L) was prepared with tetrahydrofurane extra pure (purity > 99.5%) and stabilized with 250 ppm of butylated hydroxytoluene (BHT). The polyol was dissolved (100 g/L) in dimethyl sulfoxide synthesis grade (purity > 99.5%), and the isocyanate solutions (44 g/L) were made with a mixture of acetonitrile HPLC grade (purity > 99.9%)/tetrahydrofurane in a relation of 65/35% vol., respectively. Moreover, acetone synthesis grade (purity > 99.5%) was employed for the drying step. All the solvents were provided by Scharlab, S. L (Barcelona, Spain).

Reticulated polyurethane foams were provided by Recticel Ibérica, S.L. (Barcelona, Spain).

### 2.2. Synthesis of Polyurethane Aerogels and PU_F_–PU_A_ Composites

**Figure 1:**
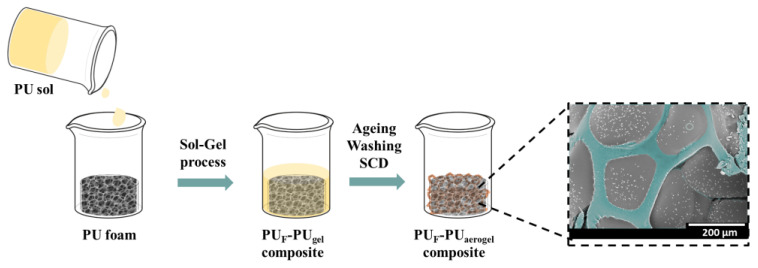
PU_F_–PU_A_ synthesis procedure.

The polyurethane foam–polyurethane aerogel composites were synthesized through a double step as follows (Figure 1). First, the corresponding amount of the pentaerythritol solution was added to the p-MDI solution (iso/pol molar ratio of 2.32). After the addition of a 4 wt.% (calculated from the total mass of polyol and isocyanate) of catalyst, the blend was stirred with a EUROSTAR Power control-visc P1, IKA (Artisan Technology Group, Champaign, IL, USA), provided with a 50 mm diameter Lenart disc at 500 rpm for 20 s, as previously described by the authors in [20,21]. Finally, the solution was poured into a plastic container where, in the case of the composites, the corresponding reticulated foam had been previously placed, until it was completely covered. Once the gelation time was reached (around 28 min), the obtained gel-like composites were covered with acetonitrile for 24 h at room temperature. After this time, the composites were demolded and washed twice (each time within 24 h and at room temperature) with acetonitrile to remove residual compounds.

Finally, the PU_F_–PU_A_ gels were covered with acetone to prevent preliminary solvent evaporation and were dried under supercritical conditions with CO_2_ at 100 bar and 40 °C.

### 2.3. Characterization Techniques

#### 2.3.1. Density, Shrinkage, and Aerogel Mass

The densities of the foams, reference aerogel, and PU_F_–PU_A_ composites were measured by using a AT261 MettlerToledo (Columbus, OH, USA) balance for measuring the mass and a caliper (0.01 mm resolution) for the geometric volume, as indicated in ASTM D1622/D1622M-14 [24].

The volumetric shrinkage (*S_υ_*) of the composites was measured as the relationship between the volume of the final composites (*V*) and the volume of the initial foam (*V*_0_), as described by Equation (1):

(1) $$S_{v}\left(\%\right)=\left(1-\frac{V}{V_{0}}\right)\times 100$$

The aerogel porosity (Π) was calculated by Equation (2):

(2) $$\Pi =\left(1-\rho_{r}\right)\times 100$$

where *ρ_r_* is the relative density calculated by dividing the aerogel density by the solid polyurethane density.

For the PU_F_–PU_A_ composites, the percentage of polyurethane aerogel mass included into the polyurethane foams is given by the following equation:

(3) $$aerogel\;mass\;\left(\%\right)=\frac{\left(m_{composite}-m_{foam}\right)}{m_{composite}}\times 100$$

where *m_composite_* and *m_foam_* are the masses of the composite and initial polyurethane foam.

#### 2.3.2. Nitrogen Adsorption–Desorption

Nitrogen sorption was employed for measuring the specific surface area (SBET) of the reference polyurethane aerogel, by the Brunauer–Emmet–Teller (BET) method through a Micromeritics (Norcross, GA, USA) ASAP 2020 instrument at the University of Malaga (Malaga, Spain). First, the sample was degassed in vacuum at 50 °C for 24 h. Through the desorption branch of the isotherm, the corresponding pore size was calculated by Barrett–Joyner–Halenda (BJH) [25] method through the Equation (4):

(4) $$\Phi_{p\;}=\frac{4V_{p}}{S_{BET}\;}$$

where *S_BET_* is the surface area (m^2^/g) and *V_p_* the pore volume calculated as the difference between the total volume and the volume of the solid phase:

(5) $$V_{p}=\frac{1}{\rho }-\frac{1}{\rho_{S}}$$

#### 2.3.3. Scanning Electron Microscopy (SEM)

The polyurethane aerogel was metalized through an iridium sputter coater (EMITECH (Fall River, MA, USA) K575X Sputter Coater) and the micrographs were obtained with an ESEM Scanning Electron Microscope (QUANTA 200 FEG, Hillsboro, OR, USA).

The polyurethane foams were metallized with gold instead of iridium owing to the significantly higher pore size. These samples were visualized by a scanning electron microscope (FlexSEM 1000, Hitachi (Tokio, Japan)) and their cells were measured by a software based on Image J/FIJI [26].

The PU_F_–PU_A_ composites were observed by a scanning electron microscope (FlexSEM 1000, Hitachi, Tokio, Japan) with a BackScattered Electron Detector (BSE).

#### 2.3.4. Mechanical Properties

Mechanical tests were carried out for the pure polyurethane foams, for the reference polyurethane aerogel, and for the manufactured PU_F_–PU_A_ composites. A universal testing machine (Instron model 5500R6025 (Norwood, MA, USA)) was used to perform compression–decompression tests with a load cell of 1 kN at a rate of [height/10] mm/min for both displacements, following the ASTM D1621-00 test [27]. Tests were performed on cylindrical samples of 15–20 mm in diameter and 10–12 mm in height, at ambient conditions (23 ± 2 °C and 50 ± 10% relative humidity as indicated by ISO 291:2005 [28]).

Five compression–decompression tests were performed until reaching 10% of strain in order to assess the recovery ability of the samples. The decompression test followed the compression test without any time between them. The energy loss coefficient (*ELC*) was obtained from the hysteresis loop area by using the Equation (6) [29]:

(6) $$ELC\;\left(\%\right)=\frac{A_{L}-A_{U}}{A_{L}}\times 100$$

where *A_L_* and *A_U_* are the areas under the loading and unloading curve, respectively.

The elastic modulus was calculated from the linear region of the stress–strain curves, as well as the stress at different selected strains (σ_10%_, σ_25%_, σ_50%_, and σ_75%_). A preload of ca. 0.5 kPa was applied for all the experiments.

#### 2.3.5. Thermal Conductivity

The thermal conductivity of either the PU aerogels and the resultant composites were measured by a thermal heat flow meter FOX 314 (TA Instruments/LaserComp, Inc. (New Castle, DE, USA)) according to the standards ASTM C518 [30] and ISO 8301 [31].

PU foams were obtained as large plane sheets, allowing them to be measured with the transducer of the thermal heat flow meter. Since the sample dimensions for the composites are smaller than the heat flux transducer area, an external heat flux sensor gSKIN^®^ XM 27 9C (greenTEG AG, Rümlang, Switzerland) (4.4 × 4.4 mm^2^) was used together with a data logger gSKIN^®^ DLOG-4219 (greenTEG AG, Rümlang, Switzerland) [32]. The temperature gradient was applied using the heat flow meter FOX 314. Instead of using the plate temperatures for the calculations, the sample temperature was monitored during the measurements by two thermocouples that were in contact with the upper and lower surfaces of the specimen. The cylinder dimensions were ca. 30 mm in diameter and 12 mm in height.

All the samples were measured at four average temperatures: 10, 20, 30, and 40 °C, with the hot and cool plates being 10 °C above and below the selected temperature, respectively.

## 3. Results and Discussion

The polyurethane foams and a pure aerogel were characterized as references prior to the PUF–PUA composite synthesis and characterization. Their densities, pore size, thermal conductivities, and mechanical behavior were analyzed. Once these materials were assessed, the manufactured composites were fully characterized, and their properties were compared with those of the reference materials.

### 3.1. Polyurethane Foams and Polyurethane Aerogel Characterization

**Figure 2:**
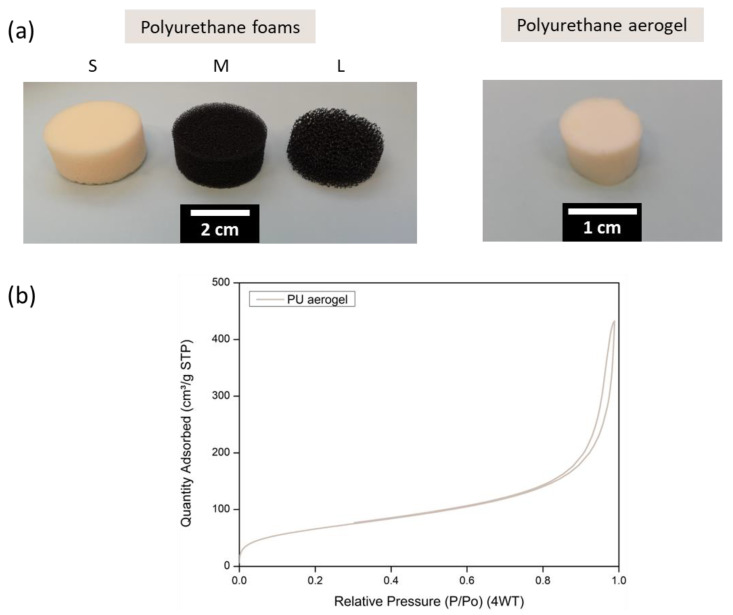
(**a**) Reticulated polyurethane foams (**left**) and polyurethane aerogel (**right**). (**b**) Nitrogen sorption quantity at each relative pressure for the polyurethane aerogel.

For this work, different polyurethane foams were selected that were characterized by an open-porous structure whose cells are defined by polymeric struts (Figure 2a). The main properties of these foams can be found in Table 1. All the reticulated foams employed present a similar density of ca. 29 kg/m^3^. Their pore sizes are differ greatly, with the average value of the smallest one being 0.4 mm, then 1.4 mm, and the largest average pore size being 4.3 mm. According to the pore size, we labelled the samples as: S (small), M (medium), and L (large), respectively. The thermal conductivities are in agreement with the pore size, being higher for larger pores due to the rise of the radiative contribution and the potential contribution of the convection mechanism for the materials with larger pores. The thermal conductivities of the samples at 10 °C range from 34 to 50 mW/(m K) (Table 1).

A reference polyurethane aerogel was synthesized as described in the Experimental Section. After the corresponding CO_2_ supercritical drying, the measurement of the aerogel bulk density gave a value of 129 kg/m^3^, which implies a high porosity of 89% (taking as solid density a value of 1170 kg/m^3^ [23]). Its textural properties were analyzed by means of nitrogen sorption (Figure 2b), reaching a high internal surface area of 242 m^2^/g and a pore size of ca. 114 nm. These features significantly reduce the effective thermal conductivity to values of 13.90 mW/(m K). The relatively low density helps to decrease the conduction through the solid phase. In addition, a high phonon scattering in the small connections between the spherical polymeric particles is expected. Additionally, the small size of the pores, of a few nanometers in diameter, promotes the Knudsen effect [33], decreasing the gaseous conduction through the aerogel.

### 3.2. PU_F_–PU_A_ Composites Characterization

**Figure 3:**
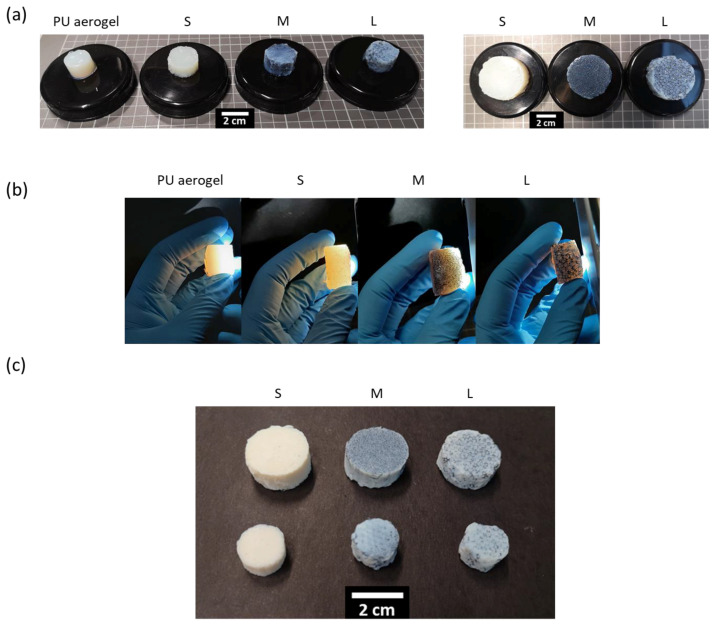
(**a**) PU reference gel and PU_F_–PU_A_ gels used for the mechanical tests (**left**) and thermal conductivity measurements (**right**). (**b**) Gels’ appearance when a light beam passes through the sample. (**c**) PU_F_–PU_A_ dried composites.

Polyurethane foam/polyurethane aerogel composites were fabricated by a sol–gel process as described in the Experimental Section. Examples of the PU_F_–PU_A_ gel composites obtained are depicted in Figure 3a. Their reticulated structure can be differentiated when light passes through them owing to the gel transparency [23,34], as observed in Figure 3b. This supports the notion that the foam pores are completely filled with the aerogel, which forms a continuous network with the polymeric skeleton of PU foam.

Examples of the PU_F_–PU_A_ composites obtained once gels were dried by supercritical CO_2_ are shown in Figure 3c. Once dried, the aerogels slightly lose some transparency, turning to a whitish blue color. Nevertheless, the foam structure is still appreciated, thus accounting for the good interaction between both matrices.

#### 3.2.1. Density and Aerogel Mass

The composites’ bulk density was measured, and the percentage of aerogel mass was calculated from the relationship between the initial mass of the PU foam and that obtained for the PU_F_–PU_A_ composite. Moreover, the volumetric shrinkage (S_v_) was assessed for all the composites. These results are gathered in Table 2.

As expected, the bulk density of the composites is higher than that of the foams because of the aerogel incorporation. However, the densities of the composites are lower than the expected values for a PU aerogel of 129 kg/m^3^. This can be explained by the shrinkage experimented on the composites during the manufacturing steps which is significantly lower than that of the monolithic aerogel (66.3%). Therefore, the density of the aerogel when being incorporated into the PU skeleton is reduced and the shrinkage is strongly decreased. To evaluate the density of the PU aerogel inside the composites, Equation (7), obtained from the rule of mixtures, was applied:

(7) $$\rho_{aerogel}=\frac{\rho_{composite}-\left(\rho_{{rPU}_{foam}}\cdot \;\rho_{{rPU}_{solid}}\right)}{\Pi_{{PU}_{foam}}}\;$$

where *ρ_rPU foam_* is the relative density of the PU foam, accounting for the solid skeleton of polyurethane, *ρ_PU solid_* is the density of solid PU, *Π_PU foam_* is the porosity of the polyurethane foam accounting for the pore volume occupied by the aerogel, and *ρ_aerogel_* is the aerogel density inside the composites. The obtained values are found in Table 2. It is noticeable that the aerogel density is lower due to the skeleton which prevents the shrinkage to a high extent. This prevention is higher for larger pores, reaching the lowest aerogel density of 97.6 kg/m^3^ instead of 129 kg/m^3^ and becoming the first advantage of this reinforcement strategy. Additionally, the aerogel porosity increases from 88% to 91% as a consequence of the effect of the PU skeleton.

Considering foam pore size, bulk density follows a clear trend: smaller pores lead to denser composites. This tendency accounts for the volumetric shrinkage of the samples. Thus, the S composite shows a stronger shrinkage and the highest density (134 kg/m^3^). The volumetric shrinkage of the M composite is slightly lower (8.9%) and, therefore, its density is lower as well, which reaches a value of 125 kg/m^3^. Finally, the composite with the largest pores (L composite) shows a negative shrinkage value of −1.6%. This is mainly due to the swelling that the PU foams make when submerged into the sol. For this reason, since the L composite swells while its shrinkage is not significant, the sample with the lowest density value of 123 kg/m^3^ is obtained.

The amount of aerogel which is able to fill the pore structure is not affected by the pore size, since all of the composites are composed by ca. 80 wt.% of polyurethane aerogel.

#### 3.2.2. Porous Structure

**Figure 4:**
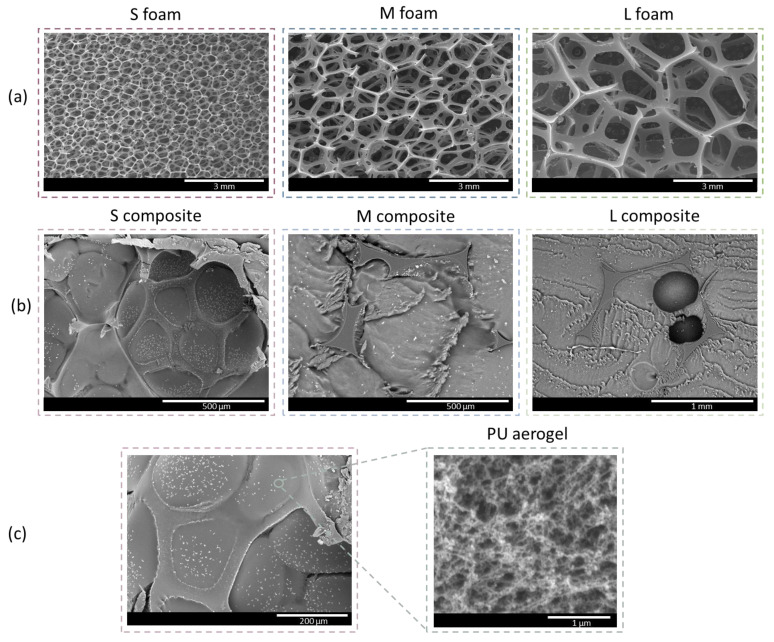
(**a**) Cellular structures of the reticulated foams, (**b**) morphology of the PU_F_–PU_A_ composites, (**c**) polyurethane aerogel contained inside the foam pores.

Figure 4a shows the cellular structure of the foams formed only by a network of polymeric struts, as well as the substantial difference between the pore size of the foams. The polyurethane aerogel is able to fill the foam pores depending on both the interaction between both matrices and the viscosity of the sol before the gelation step, being key factors in the composite formation.

Figure 4b shows the micrographs for the PU_F_–PU_A_ composites. The cell of the S composite (formed by the smallest pore-sized foam) is clearly filled with the polyurethane aerogel. In the composites with larger pore sizes (M and L), almost all the polyurethane struts are covered by aerogel, as finding a region in which foam is visible is rather difficult. These micrographs show a high cohesion between the aerogel and the initial polyurethane foam.

Finally, the magnification of one of the pore walls, displayed in Figure 4c, shows the inner aerogel structure composed by nanometric and spherical particles assembled through thin necks. The spherical shape of these particles, apart from leading to a high internal surface area, helps to optimize the contact with the polyurethane foam and, therefore, to extend the interactions between the polyurethane groups of the aerogel and the foam. These interactions can be based on hydrogen bonding between the urethane and the isocyanurate rings [35].

Figure S1 (Supporting Information) contains the scanning electron micrographs of the PU aerogels included inside the foam pores, as well as the monolithic aerogel at different magnifications. The decrease in the aerogel density when included into the reticulated PU structure contributes to the increase in the porosity of these aerogels.

#### 3.2.3. Mechanical Properties

##### Elasticity

**Figure 5:**
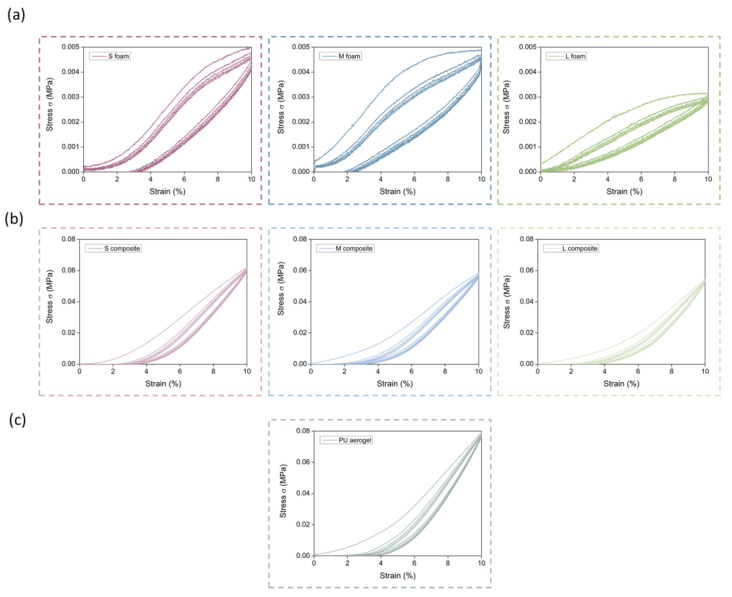
Compression–decompression curves for (**a**) polyurethane foams, (**b**) PU_F_–PU_A_ composites, (**c**) polyurethane aerogel at a strain of 10%.

**Figure 6:**
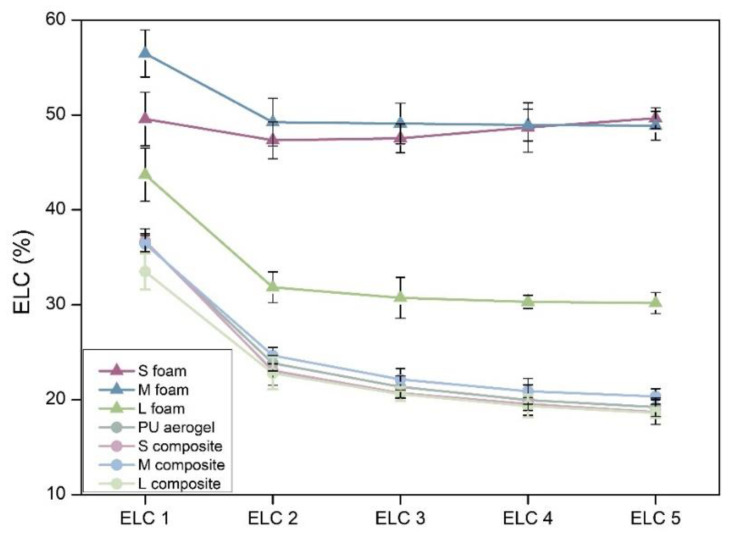
ELC graph the produced samples.

The results for the compression–decompression tests for all the materials are plotted in Figure 5 and the hysteresis have been assessed, obtaining the energy loss coefficient for each cycle (Figure 6). The S, M, and L foams (Figure 5a) show a remarkably flexible behavior. Nevertheless, a notable difference on the hysteresis of these samples is evident. The L foam shows the lowest stiffness and the smallest hysteresis area, meaning that a smaller amount of energy is dissipated during the experiment and, therefore, its elasticity is the highest (recovering the initial height).

Comparing these results with those of the composites (Figure 5b), a completely different behavior is observed. The stress applied to obtain a deformation of 10% is ca. 12 times higher than that of the corresponding PU foams (values from ca. 0.005 MPa to 0.06 MPa). When comparing the compressive–decompressive behavior obtained for the composites (Figure 5b) with that of the pure PU aerogel (Figure 5c), a similar recovery ratio was observed. However, the pure aerogel showed a higher strength than those of the composites, reaching a higher stress of 0.08 MPa for a 10% of strain.

Moreover, the hysteresis areas for the S, M, and L composites are significantly smaller than those of the foams (plotted in Figure 6), accounting for the higher elasticity of these samples. It is noticeable that the obtained energy loss coefficient values for the composites are similar to those obtained for the monolithic aerogel being ca. 36%, 23%, 21%, 20%, and 19% for the five compression–decompression cycles, respectively. Therefore, the synthesized composites present a great elasticity, allowing them to reach a deformation of 10% without their properties being significantly worsened. When comparing between the composites, there is a slight decrease in the hysteresis sloop area for the composite with the largest pores (L composite), meaning that this is the more elastic sample, more than the monolithic aerogel (raw values are gathered in Supporting Information Table S1). Here is where the second advantage of these composites lies: reaching materials with an improved elasticity in comparison with the initial PU foam network.

##### Stiffness and Strength

**Figure 7:**
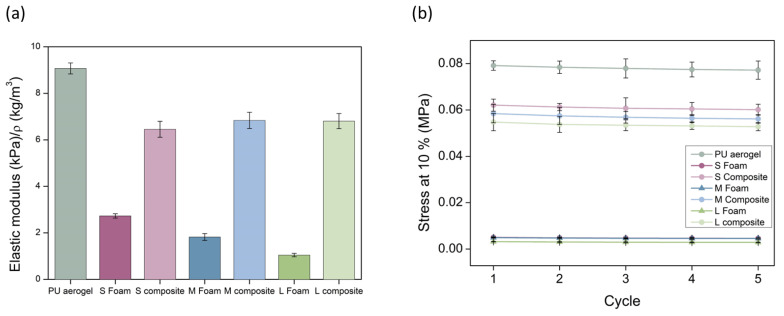
(**a**) Normalized elastic moduli for all the samples. (**b**) Stress at a strain of 10% for each compression–decompression cycle.

The elastic moduli have been calculated from the linear region of the five compression cycles and normalized with the sample density (raw values are gathered in Supporting Information Table S2). This normalization was carried out to avoid the effect of density as reported by Linul et al. for polyurethane foams [36] and by Andersons et al. for polyisocyanurate foams [37]. This procedure allows one to correct, up to some extent, the differences caused by density between samples and to compare their mechanical properties. There exist several studies in which the value to which density is powered is calculated. For instance, the Young’s modulus sensitivity on the bulk density for polyurethane aerogels was calculated by Chidambareswarapattar et al., finding values between 3.73 and 7.75 [38]. Therefore, owing to the uncertainty of this exponent, which is even larger when producing composites with different materials (foams and aerogels), the elastic moduluswas corrected with the bulk density. The obtained relative values for the first cycle are plotted in Figure 7 (left). A slight decrease on the elastic modulus in comparison with the monolithic aerogel is observed. Nevertheless, the stiffness of the PU foams has been significantly enhanced, reaching an increase of 2.4, 3.8, and 6.5 times the initial elastic modulus of the PU foams. This rigidity is due to the incorporation of a PU aerogel with a significant elastic modulus into the foam pores that were previously filled with air.

Regarding the stress at a strain of 10% reached for each of the compression–decompression cycles, different behaviors are observed. Three groups can be differentiated in Figure 7’s right graph: the polyurethane foams with the lowest stress (values ca. 0.005 MPa) are, therefore, the softest samples (numerical values are gathered in Table S3). The stress for the PU_F_–PU_A_ composites is remarkably increased, reaching values of ca. 0.06 MPa for all of them; that is, 12.4-, 12.0-, and 17.3-fold higher modulus for the S, M, and L composites, respectively. Thus, the foam with the largest pores is contributing to the increase in the strength of the composite. Finally, the PU aerogel presents the highest stress of 0.08 MPa. Therefore, foams filled with PU aerogel show intermediate mechanical properties between those of the initial foams and those of the pure aerogel, being significantly stiffer than those with air-filled pores. It must be noticed that the stress is maintained almost constant during the five cycles.

**Figure 8:**
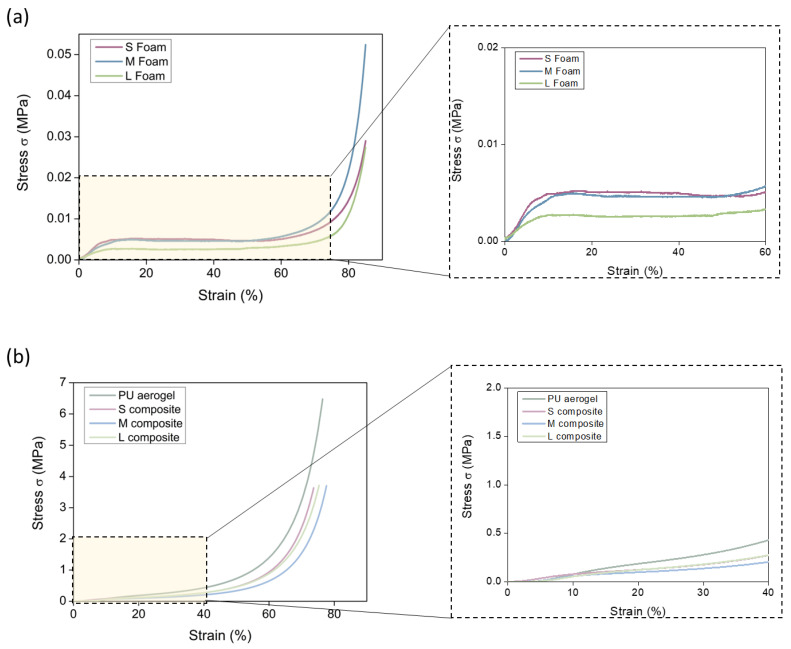
(**a**) Strain–stress curves for the polyurethane foams, (**b**) composites at deformations of 80%.

Finally, experiments at higher deformation were performed. As represented in Figure 8a, the PU foams show three different regions: an initial linear region in which the deformation is elastic, a plateau region, and the final densification region for strains higher than 70%. It is noticeable that the L foam (green color) shows a lower compressive strength than S and M foams, reaching the same strains at lower stress.

For the composites and the PU aerogel (Figure 8b), the behavior is similar to that observed for the PU foams, although the linear region ends at small strains (ca. 8%) and the values of stress are clearly higher. This linear part is followed by a plateau region comprising strains from 8 to 40% and, finally, densification progressively occurs, being sharper at 60% of deformations. Remarkably, neither the pure aerogel nor the composites are broken under high compressive stress but undergo a strong densification of particles along the aerogel pore volume.

**Figure 9:**
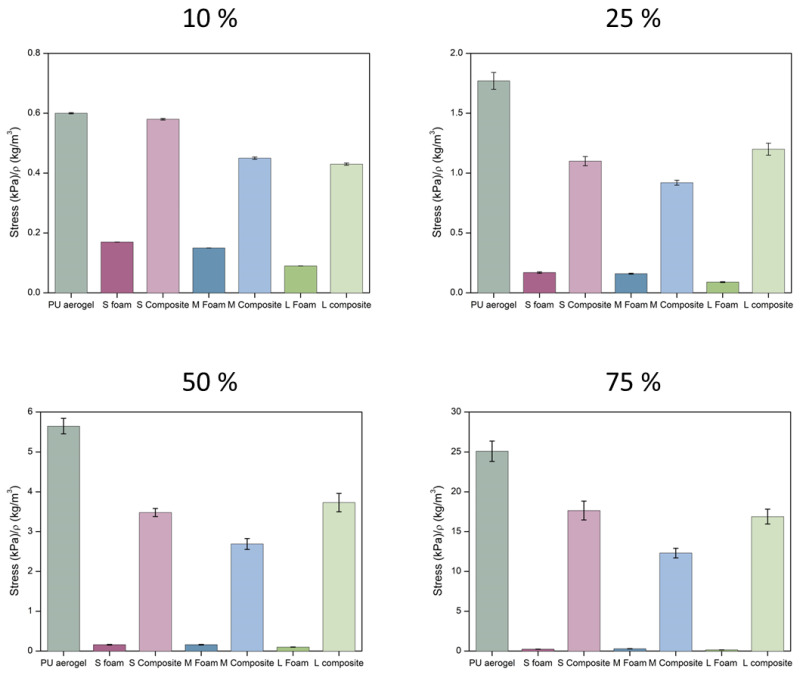
Normalized stress at different strains (10, 25, 50, and 75%) for the samples under study.

The numerical values for different strains can be extracted from these curves and normalized by the bulk density in order to stablish a comparison between samples (Figure 9). The numerical values can be found in Table S4. For all the assessed strains, the stress needed for the pure aerogel is slightly higher than for the composites. On the other hand, a considerable stiffness improvement can be observed when comparing the PU foams with the final composites reaching notably higher stress at all the strains (16-, 9-, and 25-fold higher for S, M, and L composites, respectively).

As expected, the composites showed an intermediate behavior between the PU aerogel and the PU foams, reaching significantly higher compressive strength that the latter. The three composite materials show similar values of the relative compressive strength as a function of the cell size of the foam at 25, 50, and 75% strain. Only for low strains is it observed that there is a trend with cell size, increasing the compressive strength when the foam used has a lower cell size.

#### 3.2.4. Thermal Conductivity

The insulating capacity of all the samples was measured by the steady state method. Four main contributions to the total thermal conductivity should be taken into account:

(8) $$\lambda_{T}=\lambda_{s}+\lambda_{g}+\lambda_{r}+\lambda_{c}$$

where *λ_s_* is the conduction through the solid phase, *λ_g_* the conduction through the gaseous phase, *λ_r_* the radiation term, and *λ_c_* the convection within the cells. For the initial polyurethane foams, the convection contribution should be considered since the pores are interconnected, promoting a relevant gas motion.

The solid contribution for the foams is based on the phonon transmission through the solid rib-skeleton, and can be described by Equation (9) [39]:

(9) $$\lambda_{s}=\rho_{r\;\left(PU\;foam\right)}\cdot f_{s}\cdot \frac{\lambda_{s}^{\prime }}{3}$$

where ρ*_r_* is the relative density, *f_s_* the mass fraction on the struts, which is equal to 1 for reticulated foams, and *λ*′*_s_* the thermal conductivity of the polymeric matrix (1160 kg/m^3^). However, when these pores are filled with aerogel, the solid contribution is expected to be different, and the phonon transfer through the spherical nanoparticles of the solid phase of the aerogel must be taken into consideration, as described by Equation (10) [40]:

(10) $$\lambda_{s}=\rho_{r\;\left(PU\;aerogel\right)}\cdot \;{\lambda^{\prime }}_{s}\cdot \frac{\upsilon }{\upsilon_{s}}$$

where sound-speed-related terms are included (*υ* is the speed of sound through the aerogel and *υ_s_* that through the solid matrix).

Hence, once the composites are synthesized, there exist two solid contributions: the one corresponding to the solid PU matrix, and the conduction through the solid phase of the polyurethane aerogel. Therefore, two different influences will affect this contribution. On the one hand, an increase in the final density would lead to a higher solid contribution. On the other hand, the small interconnections between the spherical particles through thin necks would lead to a hindered phonon transfer, thus reducing the speed of sound through the aerogel phase. This effect, together with the size-effect promoting a stronger phononic scattering [41], will contribute to a reduced solid term. As a first approximation, assuming that a series model is valid for the composites, the conduction along the solid phase for these composites could be expressed as follows:

(11) $$\lambda_{s}=[\rho_{r\;\left(PU\;foam\right)}\cdot \lambda_{s}^{\prime }]+[\Pi_{\left(PU\;foam\right)}\cdot \rho_{r\;\left(PU\;aerogel\right)}\cdot ({\lambda^{\prime }}_{s}\cdot \frac{\upsilon }{\upsilon_{s}})]\;$$

The first term is related to the contribution of the solid PU skeleton, whereas the second term, which includes the porosity of the PU foam (*Π*_(*PU foam*)_), accounts for the solid contribution of the aerogel.

The gaseous contribution for the polyurethane foam can be described by Equation (12), where the key parameters are the porosity (1 − *ρ_r_*_(*PU foam*)_) and the thermal conductivity of the gas inside the pores *λ*′*_g_* (air):

(12) $$\lambda_{g}=\left(1-\rho_{r\;\left(PU\;foam\right)}\right)\cdot \lambda_{g}^{\prime }$$

Nevertheless, once the aerogel is filling the inner gaseous phase of these foams, the gaseous contribution will be due to the gas phase inside the aerogel phase. The effect occurring when the size of the material pores belongs to the nanometric scale is well-known as the Knudsen effect, described by Equation (13) [33]:

(13) $$\lambda_{g}=\Pi_{\left(PU\;foam\right)}\cdot \left(1-\rho_{r\;\left(PU\;aerogel\right)}\right)\cdot \frac{{\lambda^{\prime }}_{g0}\left(T\right)}{1+\frac{2\beta l_{g}}{\phi_{pore}}}$$

where *λ*′*_g_*_0_ is the thermal conductivity of the gas inside the pores, *β* is the correlation factor for the energy transfer between the aerogel structure and the gas molecules (1.64 for air [42]), *l_g_* is the mean free path of the gas molecules (c.a. 70 nm for air [33,43]), and *ϕ_pore_* is the average pore size. Owing to this effect, the gaseous contribution is expected to be strongly reduced, leading to more efficient insulating materials.

Finally, the radiative term *λ_r_* depends mainly on the mean temperature (*T*) as well as on the extinction coefficient (*K_e_*), as shown by the Rosseland equation [44] (Equation (14)):

(14) $$\lambda_{r}=\frac{16\cdot n^{2}\cdot \sigma \cdot T^{3}}{3\cdot (K_{e)}}$$

in which *σ* is the Stephan–Boltzmann constant (5.67 × 10 − 5 mW/m^2^ K^4^), n is the refractive index (close to 1 for low-density cellular polymers [45]), and *K_e_* is the extinction coefficient. The effective value of this parameter comprises the effect of two contributions: the absorption of the solid PU skeleton, and the absorption and scattering produced by the PU aerogel.

**Figure 10:**
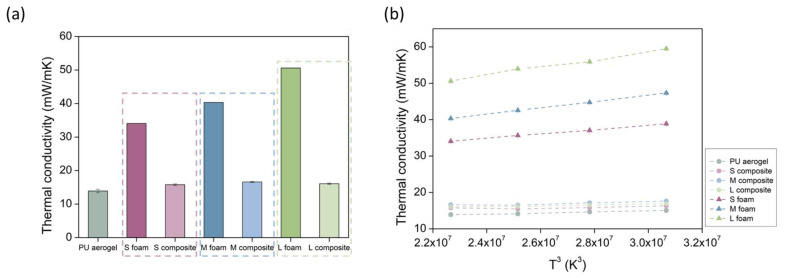
(**a**) Thermal conductivities for all the samples at 10 °C, (**b**) representation of thermal conductivity vs. T^3^.

The experimental values for the thermal conductivity obtained at 10 °C (numerical values can be found in Supporting Information Table S5), are plotted in Figure 10a. As expected, a noticeable reduction in the final thermal conductivity is observed when the polyurethane aerogel is included into the foam pores. For the foam with the smallest pores (S foam), the initial value of 34.07 mW/(m K) is decreased to 15.79 mW/(m K). For the foam with the medium pore size (M foam), the former value of 40.33 mW/(m K) is decreased to 16.61 mW/(m K), and, when the pores are larger (L foam), the reduction goes from 50.60 mW/(m K) to 16.07 mW/(m K). These values imply reductions of 53, 58, and 68% for the S, M, and L composites, respectively. This trend indicates that bigger pores lead to a more noticeable reduction in the thermal conductivity. Moreover, it is remarkable that the PU_F_–PU_A_ composites reach final insulating capacities very similar to those of the pure aerogel, with a value of 13.90 mW/(m K) at 10 °C.

The thermal conductivity values of PU foams found in the literature are comprised between 25–33 mW/(m K) [2], although slightly lower values have been reached. For example, Kurańska et al. reached values of 22.8 mW/(m K) when using lignin- and rapeseed-based polyols [46], or even ca. 20 mW/(m K) were achieved by Jarfelt et al. for microcellular foams [47]. One commonly used strategy is the use of gases with high molecular weight such as cyclopentane or hydrofluoroolefins (HFO) as blowing agents which remain retained inside the pores. Nevertheless, these values are far from those reached by the aerogel inclusion strategy, which leads to a more effective insulating performance.

Regarding the polyurethane aerogels reported in the literature, the thermal conductivity values are ranged between 12 and 103 mW/(m K) depending on the main characteristics of the samples. Thus, the values reached by these PU_F_–PU_A_ composites are comparable to some of the PU monolithic aerogels reported in the literature [48].

According to the Equation (14) for the radiative term, the thermal conductivity–temperature dependence is to the third power. For this reason, the influence of the temperature on the thermal conductivity is displayed in Figure 10b, by plotting λ_t_ vs. T^3^. There is a stronger dependence of the thermal conductivity with the measurement temperature for the initial foams. Nevertheless, for the PU aerogel and composites, this influence is weaker since the absorption of these materials is higher and, therefore, the extinction coefficient increases, contributing to a reduction in the radiative term.

Through the use of the previous equations, the theoretical contributions to the effective thermal conductivity can be obtained for the PU foams, monolithic aerogel, and composites in order to understand which terms are strongly modified when the composite is synthesized. The gaseous contribution was calculated by the use of the Equations (12) and (13) for the foams and composites, respectively. In the case of the PU foams, due to the difficulty of calculating the convective contribution, the sum of the radiation and convection terms was obtained by subtraction of the gaseous and solid terms from the effective thermal conductivity.

**Figure 11:**
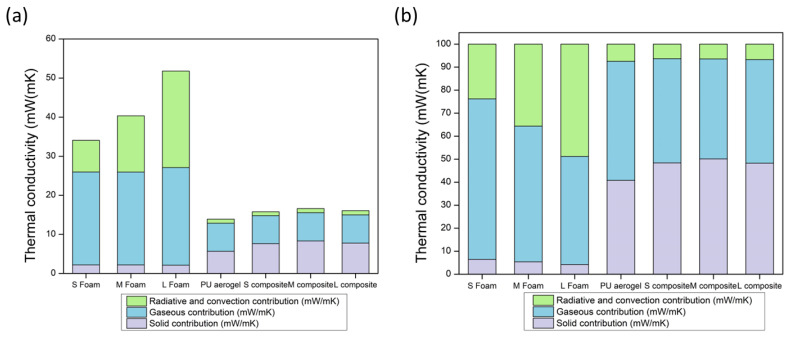
(**a**) Thermal conductivity contributions for the PU foams, PU aerogel, and composites, (**b**) percentage of the thermal conductivity contributions for the PU foams, PU aerogel, and composites.

The solid contribution was estimated by the Equation (9) for the PU foams, and as the difference between the effective thermal conductivity and the gaseous and radiative (Equation (14)) terms for the aerogel and composites due to the unknown value of the speed of sound on these materials, which will be further studied in future works. The obtained results are plotted in Figure 11.

There is a clear difference in the contributions’ distribution. For the foams, the radiative and convective terms have a relevant weight owing to the large pores forming their structures. Nevertheless, for the aerogel, this contribution is minimized due to the high absorption coefficients that aerogels and composites present and, therefore, the radiation absorption will be notably high. The gaseous contribution is the main contribution to the total thermal conductivity for foams and aerogel. However, for the foams, this term represents about the 50–70%, being, in total, ca. 24 mW/(m K), whereas for the aerogel and composites it is between 40–50%, with a value of ca. 7 mW/(m K). The explanation is based on the already explained Knudsen effect that is only present when the size of pores is significantly reduced, as occurred for the aerogel. Finally, the foams’ solid contribution is not as relevant as for the aerogel and composites owing to the fact that these reticulated foams are composed by a network of thin struts of only 2.5% of the total volume, and to the fact that the structure of the monolithic aerogel and composites is completely different. The latter show an 11% solid phase (porosities around 89%) whose structure is formed by nanometric-interconnected particles through small necks where the phonons can be transferred. Thus, the solid term for the monolithic aerogel and composites contributes 40–50% to the total thermal conductivity (ca. 5–8 mW/(m K)).

To conclude, taking into account the previous analysis, the PU_F_–PU_A_ composites were expected to show a significant reduction in the convection, radiative, and gaseous terms, promoting the consequent global thermal conductivity reduction, as demonstrated by the experimental data.

## 4. Conclusions

Polyurethane foam/polyurethane aerogel (PU_F_–PU_A_) composites have been successfully synthesized through a single-step sol–gel method and a subsequent supercritical drying procedure. The polyurethane gels are formed inside a reticulated skeleton by effectively filling the whole structure.

Densities ranging from 134 to 123 kg/m^3^ are obtained. Since the amounts of aerogel inside the composites are very similar (ca. 80 wt.%), the effect of the foam pore size on the final density is due to the aerogel shrinkage during drying, which is stronger for reduced pores. Moreover, when calculating the density of the PU aerogel inside the composites, its density has been strongly reduced due to the notably lower shrinkages when the reticulated foam is acting as scaffold. Therefore, their porosity is increased from 88 to 91%.

Scanning electron micrographs, carried out with the aim of assessing the effectivity of the interaction between the polyurethane foam and aerogel, show that all the composites are constituted by a continuous aerogel network enclosed in the foam pores, thus accounting for a remarkable cohesion between both matrices.

All the synthesized composites show a great flexibility, elasticity, and recovery under compression. The energy loss coefficient shows a slight reduction for the L composite, reaching an improved elastic behavior. When comparing the stress applied for reaching a deformation of 10% with those of the initial foams, a 12-fold higher stress is needed, accounting for their higher strength of the composites. Moreover, the PU_F_–PU_A_ composites do not break at high deformations, being densified from a 60% of strain.

Thermal conductivity: strongly decreases when the aerogel is inside the foams, reaching a reduction of the 53, 58, and 68% for the composites S, M, and L, respectively. The thermal conductivity values reached for the composites are 15.79, 16.61, and 16.07 mW/(m K) at 10 °C, thus showing a superinsulating performance. The main reason for this strong decrease of the thermal conductivity is the reduction of the gas phase and radiation contributions.

## Figures and Tables

**Table 1:** Characteristics of PU foams and PU aerogel.

Sample	Density (kg/m^3^)	SBET (m^2^/g)	φ	Λ (mW/m K)
PU aerogel	129 ± 1.5	242.1 ± 12.1	114 nm	13.90 ± 0.54
S foam	29.4 ± 0.7	-	0.44 mm	34.07 ± 0.01
M foam	29.2 ± 0.4	-	1.4 mm	40.33 ± 0.01
L foam	28.5 ± 0.5	-	4.3 mm	50.60 ± 0.01

**Table 2:** Densities, aerogel mass, and volumetric shrinkage of the PU_F_–PU_A_ composites.

Sample	Density (kg/m^3^)	Aerogel Mass (%)	S_v_ (%)	Aerogel Density (kg/m^3^)	Aerogel Porosity (%)
PU aerogel	129 ± 1.5	-	66.3	-	88.87
S foam	29.4± 0.7	-	-	-	-
S composite	134 ± 0.3	81.95	9.6	107.9 ± 5.3	90.70
M foam	29.2 ± 0.4	-	-	-	-
M composite	125 ± 1.9	80.90	8.9	99.0 ± 4.7	91.46
L foam	28.5 ± 0.5	-	-	-	-
L composite	123 ± 0.9	80.78	−1.6	97.6 ± 4.2	91.59

## Data Availability

Not applicable.

## Supplementary Materials

The following supporting information can be downloaded at: https://www.mdpi.com/article/10.3390/nano12132232/s1, Table S1. Energy loss coefficients for all the samples. Table S2: Elastic moduli for the produced samples. Table S3: Stress at a strain of 10% for each of the compression–decompression cycle and each sample. Table S4: Stress at different strains normalized by the sample density for all the samples under study. Table S5: Thermal conductivity at different measurement temperatures (10, 20, 30, and 40 °C) for all the samples. Figure S1: SEM micrographs of the nanostructure of the PUF-PUA composites and the monolithic PU aerogel.
