# Supplementary material for: Improving the Insulating Capacity of Polyurethane Foams through Polyurethane Aerogel Inclusion: From Insulation to Superinsulation
Source: Nanomaterials (Basel). 2022 Jun 29;12(13):2232. doi: 10.3390/nano12132232 (PMC9268151; doi:10.3390/nano12132232)
Supplement: Supplementary file 1 [file nanomaterials-12-02232-s001.zip › nanomaterials-1771717-supplementary.pdf]

Supporting Information

Improving the Insulating Capacity of Polyurethane Foams through Polyurethane Aerogel Inclusion: From Insulation to Superinsulation

Beatriz Merillas [1,*], Fernando Villafañe [2] and Miguel Ángel Rodríguez-Pérez [1,3,*]

[1] Cellular Materials Laboratory (CellMat), Condensed Matter Physics Department, Faculty of Science,
University of Valladolid, Campus Miguel Delibes, Paseo de Belén 7, 47011 Valladolid, Spain;
marrod@fmc.uva.es

[2] GIR MIOMeT-IU Cinquima-Química Inorgánica, Faculty of Science, University of Valladolid,
Campus Miguel Delibes, Paseo de Belén 7, 47011 Valladolid, Spain;
fernando.villafane@uva.es

[3] BioEcoUVA Research Institute on Bioeconomy, University of Valladolid,
47011 Valladolid, Spain

* Correspondence: b.merillas@fmc.uva.es (B.M.); marrod@fmc.uva.es (M.Á.R.-P.);
Tel.: +34-98-3423194 (B.M.); +34-98-3184035 (M.Á.R.-P.)

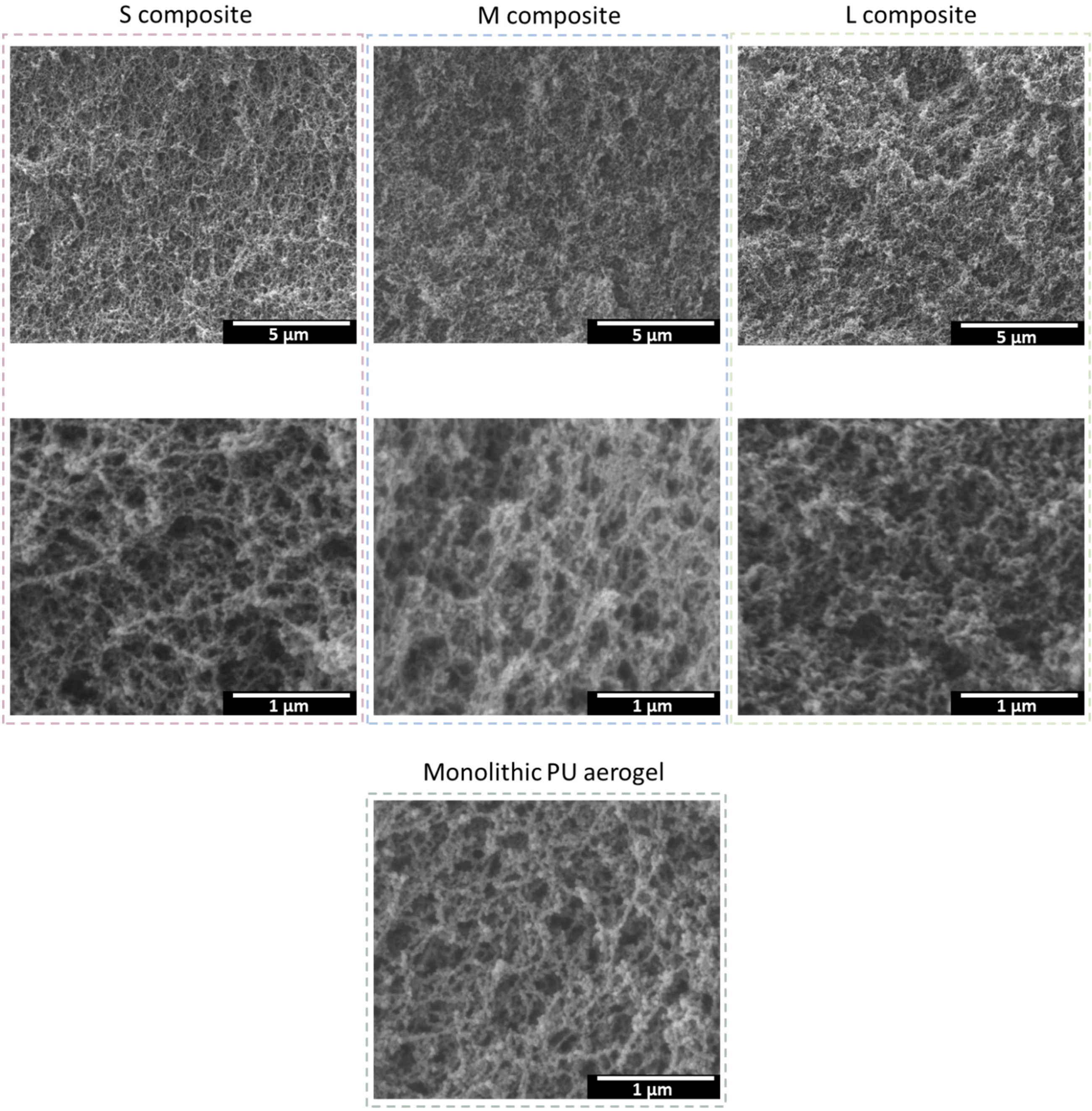

Figure S1. SEM micrographs of the nanostructure of the PUF-PUA composites and the monolithic PU aerogel.

Table S1. ELC values the produced samples

Sample	ELC 1	ELC 2	ELC 3	ELC 4	ELC 5
**S foam**	49.58 ± 2.83	47.34 ± 1.94	47.55 ± 1.52	48.69 ± 2.60	49.67 ± 1.09
**M foam**	56.49 ± 2.48	49.25 ± 2.51	49.10 ± 2.15	48.95 ± 1.66	48.88 ± 1.51
**L foam**	43.72 ± 2.80	31.85 ± 1.62	30.74 ± 2.15	30.32 ± 0.70	30.19 ± 1.12
**PU aerogel**	36.56 ± 0.95	23.84 ± 0.81	21.36 ± 1.13	19.97 ± 1.59	19.22 ± 0.98
**S composite**	36.73 ± 1.28	23.08 ± 1.55	20.70 ± 0.58	19.54 ± 0.66	18.71 ± 1.31
**M composite**	36.43 ± 0.87	24.66 ± 0.86	22.15 ± 1.13	20.90 ± 1.36	20.35 ± 0.81
**L composite**	33.51 ± 1.91	22.79 ± 1.69	20.59 ± 0.72	19.33 ± 1.18	18.62 ± 0.54

Table S2. Elastic moduli for the produced samples.

Cycle	PU aerogel	S Foam	S Composite	M Foam	M Composite	L Foam	L composite
	Elastic modulus (MPa)						
**1**	1.17	0.08	0.87	0.05	0.86	0.03	0.84
**2**	1.35	0.08	1.07	0.05	1.00	0.04	0.94
**3**	1.39	0.08	1.10	0.05	1.02	0.04	0.95
**4**	1.41	0.08	1.11	0.05	1.04	0.04	0.95
**5**	1.42	0.08	1.12	0.05	1.05	0.04	0.95

Table S3. Stress at a strain of 10 % for each of the compression-decompression cycle and each sample.

Stress at 10% of strain							
	**PU aerogel**	**S Foam**	**S Composite**	**M Foam**	**M Composite**	**L Foam**	**L composite**
**Cycle**	**MPa**						
1	0.079	0.005	0.062	0.005	0.058	0.003	0.055
2	0.078	0.005	0.061	0.005	0.057	0.003	0.054
3	0.078	0.005	0.061	0.005	0.057	0.003	0.053
4	0.077	0.005	0.060	0.005	0.056	0.003	0.053
5	0.077	0.005	0.060	0.005	0.056	0.003	0.053

Table S4. Stress at different strains normalized by the sample density for all the samples under study.

	**PU aerogel**	**S Foam**	**S Composite**	**M Foam**	**M Composite**	**L Foam**	**L composite**
**Strain (%)**	**Stress (kPa)/ϱ (kg/m³)**						
10	0.60	0.17	0.58	0.15	0.45	0.09	0.43
25	1.77	0.17	1.10	0.16	0.92	0.09	1.20
50	5.65	0.16	3.48	0.16	2.69	0.10	3.73
70	25.10	0.24	17.65	0.29	12.31	0.15	16.89

Table S5. Thermal conductivity at different measurement temperatures (10, 20, 30 and 40 °C) for all the samples.

Sample	Temperature (°C)	Thermal conductivity (mW/mK)	SD (mW/mK)
PU aerogel	10	13.90	0.54
	20	14.13	0.56
	30	14.66	0.54
	40	15.05	0.49
S foam	10	34.07	0.01
	20	35.66	0.01
	30	37.05	0.02
	40	38.88	0.01
S composite	10	15.79	0.26
	20	15.45	0.12
	30	15.84	0.13
	40	16.32	0.10
M foam	10	40.33	0.00
	20	42.57	0.00
	30	44.76	0.01
	40	47.34	0.01
M composite	10	16.61	0.16
	20	16.51	0.13
	30	17.15	0.13
	40	17.62	0.12

L foam	10	50.60	0.00
	20	53.96	0.01
	30	55.89	0.01
	40	59.50	0.01
L composite	10	16.07	0.22
	20	16.15	0.20
	30	16.52	0.11
	40	16.93	0.20
